# Estrogen regulation of germline stem cell differentiation as a mechanism contributing to female reproductive aging

**DOI:** 10.18632/aging.103080

**Published:** 2020-04-17

**Authors:** Chonthicha Satirapod, Ning Wang, Julie A. MacDonald, Minghan Sun, Dori C. Woods, Jonathan L. Tilly

**Affiliations:** 1 Vincent Center for Reproductive Biology, Massachusetts General Hospital, Boston, MA 02114, USA; 2 Department of Obstetrics, Gynecology and Reproductive Biology, Harvard Medical School, Boston, MA 02115, USA; 3 Department of Biology, Laboratory of Aging and Infertility Research, Northeastern University, Boston, MA 02115, USA; 4 Current address: Department of Molecular and Integrative Physiology, University of Kansas Medical Center, Kansas City, KS 66160, USA; 5 Current address: Department of Medical Oncology Dana-Farber Cancer Institute and Harvard Medical School, Boston, MA 02115, USA

**Keywords:** aging, estrogen, germline stem cell, oogenesis, oocyte

## Abstract

Progressive loss of ovarian estrogen (E2) production is a hallmark feature of, if not a driving force behind, reproductive aging and the menopause. Recent genetic studies in mice have shown that female germline or oogonial stem cells (OSCs) contribute to maintenance of adult ovarian function and fertility under physiological conditions through support of *de-novo* oogenesis. Here we show that mouse OSCs express E2 receptor-α (ERα). In the presence of E2, ERα interacts with the *stimulated by retinoic acid gene 8* (*Stra8*) promoter to drive *Stra8* expression followed by oogenesis. Treatment of mice with E2 *in vivo* increases *Stra8* expression and oogenesis, and these effects are nullified by *ERα* (*Esr1*), but not *ERβ* (*Esr2*), gene disruption. Although mice lacking ERα are born with a normal quota of oocytes, ERα-deficient females develop premature ovarian insufficiency in adulthood due to impaired oogenesis. Lastly, mice treated with reversible ER antagonists show a loss of *Stra8* expression and oocyte numbers; however, both endpoints rebound to control levels after ceasing drug treatment. These findings establish a key physiological role for E2-ERα signaling in promoting OSC differentiation as a potential mechanism to maintain adequate numbers of ovarian follicles during reproductive life.

## INTRODUCTION

A longstanding paradigm in reproductive biology revolved around the belief that female mammals are incapable of oogenesis after the embryonic period, such that a finite pool of oocytes enclosed within granulosa cells as follicles is set forth in the ovaries at birth [1]. As females age, this pool of follicles is then gradually depleted throughout juvenile and adult life to the point of exhaustion, leading to ovarian failure [2–4]. In women, these events culminate in the menopause, which is a time in life associated with dramatic changes in endocrine signaling in the body due in large part to a loss of ovarian follicle-derived estrogen (E2) production [5]. As a consequence, women in post-menopausal life are at increased risk for developing a diverse spectrum of health issues, ranging from hot flushes and cognitive dysfunction to osteoporosis and cardiovascular disease [6]. In mouse models of aging, interventions that sustain oocyte-containing follicle numbers in the ovaries with age have been shown to not only extend functional reproductive lifespan [7–11] but to also delay the onset of aging-associated health problems, such that many quality-of-life indices are significantly improved in females at very advanced ages [12]. Hence, under traditional thinking, the only approach for increasing ovarian lifespan would entail slowing the rate of depletion of the follicle reserve that female mammals are provided with during the perinatal period [13, 14].

The paradigm that female mammals are incapable of generating new oocyte-containing follicles during postnatal life was challenged, however, in 2004 by a study with mice reporting that adult ovaries contain proliferative germ cells which support new oocyte production to partially offset a high rate of oocyte loss through atresia [15]. Although the conclusions of this study were debated [16–21], a rare population of mitotically-active, oocyte-generating germ cells, initially termed female germline stem cells (fGSCs), was subsequently enriched from postnatal mouse ovaries and established in culture [22]. Intraovarian transplantation-based approaches showed that these isolated fGSCs could differentiate into oocytes that become follicle-enclosed, complete maturation, and fertilize to produce live offspring in natural mating trials [22]. Importantly, the method used to test the *in-vivo* functionality of fGSCs in this study [22], and the many studies that have followed repeatedly verifying the ability of transplanted fGSCs to generate viable embryos and offspring [23–29], has served as the undisputed gold standard for functional identity testing of male germline stem cells for over twenty-five years [30–32]. Nonetheless, the generation of eggs, embryos and offspring by purified fGSCs following intraovarian transplantation was still discounted by some as being insufficient to prove the existence, and functional properties, of OSCs in mammals [33], suggesting that a different bar of proof must be met in studies of males versus females [34].

Three years later, fGSCs – now referred to as oogonial stem cells (OSCs) to be consistent with the nomenclature of their male counterparts (spermatogonial stem cells or SSCs), were purified from ovarian cortical tissue of reproductive age women [24]. The cells obtained were extensively characterized for germline identity and oocyte-forming capacity [24], with outcomes that have since been independently confirmed by at least three other labs [35–38]. Consequently, several OSC-based technologies for potentially improving reproductive health and fertility are currently being explored [39–43], one of which entered clinical study with positive early outcomes reported for women seeking pregnancy through assisted reproduction [41, 44, 45]. However, other studies claiming to counter this now large body of work on mammalian OSCs have also been published [46–49], which in turn have been questioned by subsequent experiments identifying significant issues with the approaches taken [27, 50–52]. For example, Zhang et al. [46] concluded from their studies of a transgenic germline reporter mouse line that OSCs do not exist in adult mouse ovaries. Using the same methods reported in this study, two independent groups subsequently showed that OSCs can be purified from these transgenic reporter mice [27, 51] and that the purified cells are functional in terms of offspring generation [27]. Other studies have raised concerns over the validity of the purification strategies used by numerous labs to obtain OSCs from adult ovaries [48, 49], and these concerns have also been addressed in detail [29, 38, 52, 53].

One of the most pressing questions surrounding these cells related to the role, if any, that OSCs play in adult ovaries under physiological conditions [54]. Two recent studies with mice have offered important insights into this question. Using a tamoxifen-inducible system to label *POU domain class 5 transcription factor 1* (*Pou5f1*)-expressing cells in mouse ovaries, Guo et al. [55] produced clear evidence of germ cell proliferation, meiotic progression and *de-novo* oogenesis during adulthood. However, expression of *Pou5f1* in ovarian cells other than OSCs, such as oocytes [56] and resident pluripotent stem cells [57], precluded clear quantitative assessments of the number of new oocytes formed as well as fate-mapping analysis of any newly formed oocytes. These limitations were overcome in the second study, which employed two different genetic technologies – reversible suicide gene-based ablation and inducible lineage tracing [29]. To achieve this, the promoter of *stimulated by retinoic acid gene 8* (*Stra8*), a germ cell-specific gene in mice required for meiotic commitment in both sexes [58–63], was used to drive transgene expression. In addition to showing a critical need for active oogenesis during adult life in maintaining the primordial follicle pool, oocytes produced during adulthood were genetically fate-mapped in natural mating trials to the generation of viable offspring, which transmitted the transgene reporter to second-generation offspring without any discernible issues [29].

To expand on these findings and begin identification of the cues and molecular signaling pathways that regulate OSC differentiation *in vivo* in the context of ovarian aging, herein we tested if cyclic production of E2 and progesterone (P4) by the ovaries might function as a regulatory mechanism for controlling the differentiation of OSCs into oocytes during adult life. Our reasoning for this was rooted in several prior observations, the first of which is that E2 and P4 are already known to coordinate many facets of ovarian follicle formation and development [64]. Additionally, prior studies with mice have shown that the number of oocytes comprising the primordial follicle pool fluctuates during the adult reproductive cycle, with the highest numbers observed just before transition from the E2-dominant follicular phase to the P4-dominant luteal phase [65, 66]. Ovarian expression of *Stra8* is also more frequently detected in adult mouse ovaries during the follicular phase of the reproductive cycle when E2 levels are highest [67]. By combining several *in-vitro* and *in-vivo* approaches, in conjunction with a variety of genetic and pharmacologic tools to enable manipulation of E2-mediated signaling, herein we tested if E2, arguably one of the most critical hormones associated with adult ovarian function and aging-related ovarian failure, serves as a key *in-vivo* regulator of OSC differentiation and postnatal oogenesis.

## RESULTS

### Estrogen induces *Stra8* expression and oogenesis

**Figure 1:**
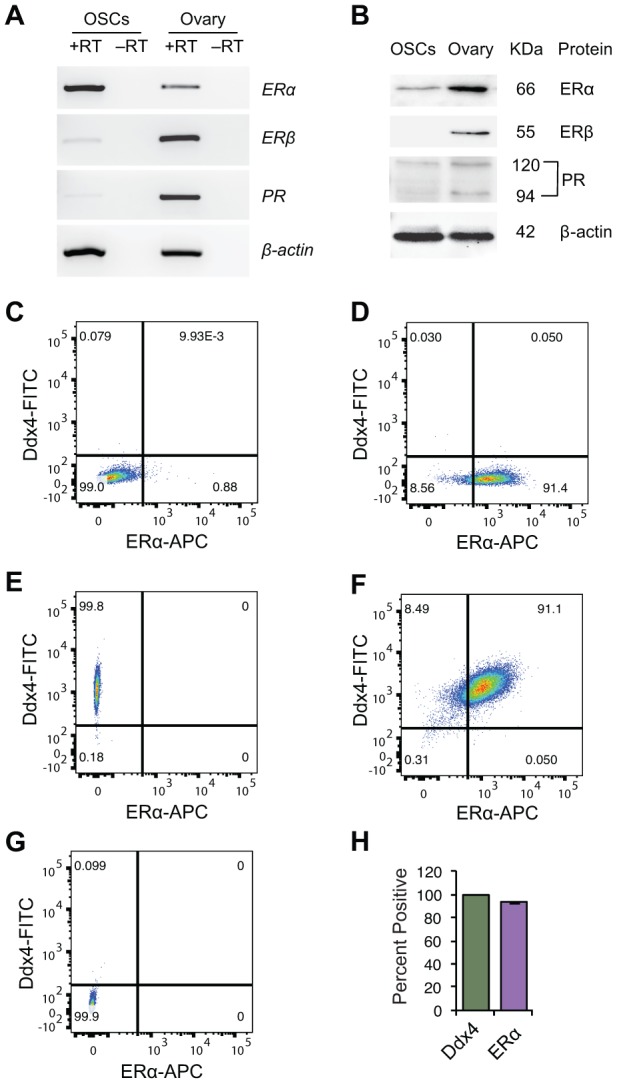
**Steroid receptor expression in purified OSCs.** (**A**, **B**) Steroid receptor expression profile in OSCs from young adult (2-month-old) mouse ovaries by RT-PCR (**A**) and western blot analysis (**B**). Expression of β-actin is shown as a control for equality of sample loading; +RT and –RT represent PCR of RNA samples with and without reverse transcription, respectively (the latter used to rule out target gene amplification from potential genomic DNA contamination). Adult ovarian tissue was used as a positive control, as indicated, since all three steroid receptors under investigation (ERα, ERβ, PR) are widely known to be expressed in this tissue. (**C**–**G**) Flow cytometric analysis of ERα protein expression in extracellular Ddx4-positive OSCs. (**C**) ERα-negative control gate; (**D**) population shift for ERα-positive cells; (**E**) population shift for extracellular Ddx4-positive cells (see panel **G** for negative control gate); (**F**) extracellular Ddx4/ERα dual-positive cells, as shown in the upper right quadrant; (**G**) extracellular Ddx4-negative control gate. (**H**) Quantification of the percent of OSCs (extracellular Ddx4-expressing cells) dual-positive for ERα expression (93.8 ± 0.5%; mean ± SEM, n = 3 independent sorts).

Using reverse transcription (RT)-polymerase chain reaction (PCR) and western blot analyses, we identified the presence of *estrogen receptor-α* (*ERα*) mRNA (Figure 1A) and ERα protein (Figure 1B), respectively, in OSCs. We also detected mRNAs encoding ERβ and PR in OSCs, but levels of these transcripts were more difficult to visualize compared with *ERα* mRNA (Figure 1A). By western blot analysis, we detected PR protein, but not ERβ protein, in OSCs (Figure 1B). Flow cytometric analysis of OSCs for co-expression of ERα and the germ cell marker, DEAD-box polypeptide 4 (Ddx4; also referred to mouse vasa homologue or Mvh), showed that over 93% of the germ cells sorted and identified as OSCs by externalized Ddx4 expression [22, 24, 29, 38, 50, 52, 53] were also ERα-positive (Figures 1C–1H).

**Figure 2:**
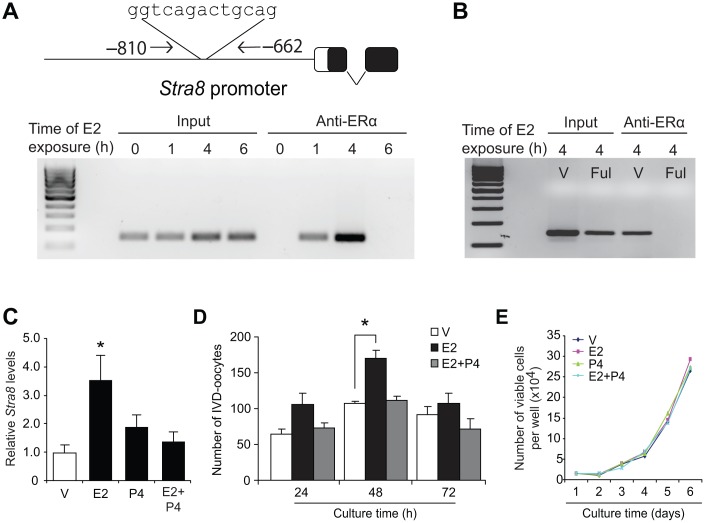
**Estrogen induces meiotic differentiation of OSCs *in vitro*.** (**A**) CHiP-PCR analysis of ERα association with a consensus ERE in the *Stra8* promoter in OSCs cultured without or with E2 (10 nM) for 1, 4 or 6 hours, using anti-ERα–based immunoprecipitation. (**B**) Confirmation of the specificity of the anti-ERα–based immunoprecipitation by pretreatment of OSCs with vehicle (V) or the pure ER antagonist, fulvestrant (Ful), prior to exposure to 10-nM E2 for 4 hours. See Figure 9A for additional data on fulvestrant. (**C**) Changes in *Stra8* mRNA levels in OSCs cultured with vehicle (V), E2 (10 nM), P4 (2 μM) or E2 plus P4 for 24 hours (mean ± SEM, n = 3 independent cultures; *P<0.05). (**D**) Number of IVD-oocytes formed by OSCs treated with V, E2, P4 or E2 plus P4 for 24, 48 or 72 hours (mean ± SEM, n = 3 independent cultures; *P<0.05). (**E**) Numbers of OSCs, seeded at an initial density of 2 X 10^4^ cells per well, through 72 hours of culture with V, E2, P4 or E2 plus P4.

To test for potential interactions of E2-activated ERα with meiotic regulatory pathways in OSCs, we next used chromatin immunoprecipitation (ChIP)-PCR assays to assess the *Stra8* promoter, which is one of the most well-defined genes in germ cell meiotic commitment [58–63]. In E2-exposed OSCs, we found that ERα occupied a consensus ER response element (ERE) in the *Stra8* promoter (Figure 2A). As a specificity control, OSCs pretreated with the pure ER antagonist, fulvestrant [68], failed to exhibit ERα interaction with the *Stra8* promoter after E2 treatment (Figure 2B). In keeping with these findings, culture of OSCs with E2 significantly increased both *Stra8* mRNA levels (Figure 2C) and *in vitro*-derived (IVD)–oocyte formation (Figure 2D), the latter serving as an established bioassay for oogenesis [24, 29, 37, 50]. While P4 treatment alone had no effect on *Stra8* expression or *in-vitro* oocyte formation, the stimulatory actions of E2 on both *Stra8* expression (Figure 2C) and oogenesis (Figure 2D) were nullified by the presence of P4. Notably, proliferation of OSCs was unaffected by treatment with either steroid alone or with a combination of the two steroids together (Figure 2E).

### Estrogen and P4 exert opposing actions on *in-vivo* oogenesis

**Figure 3:**
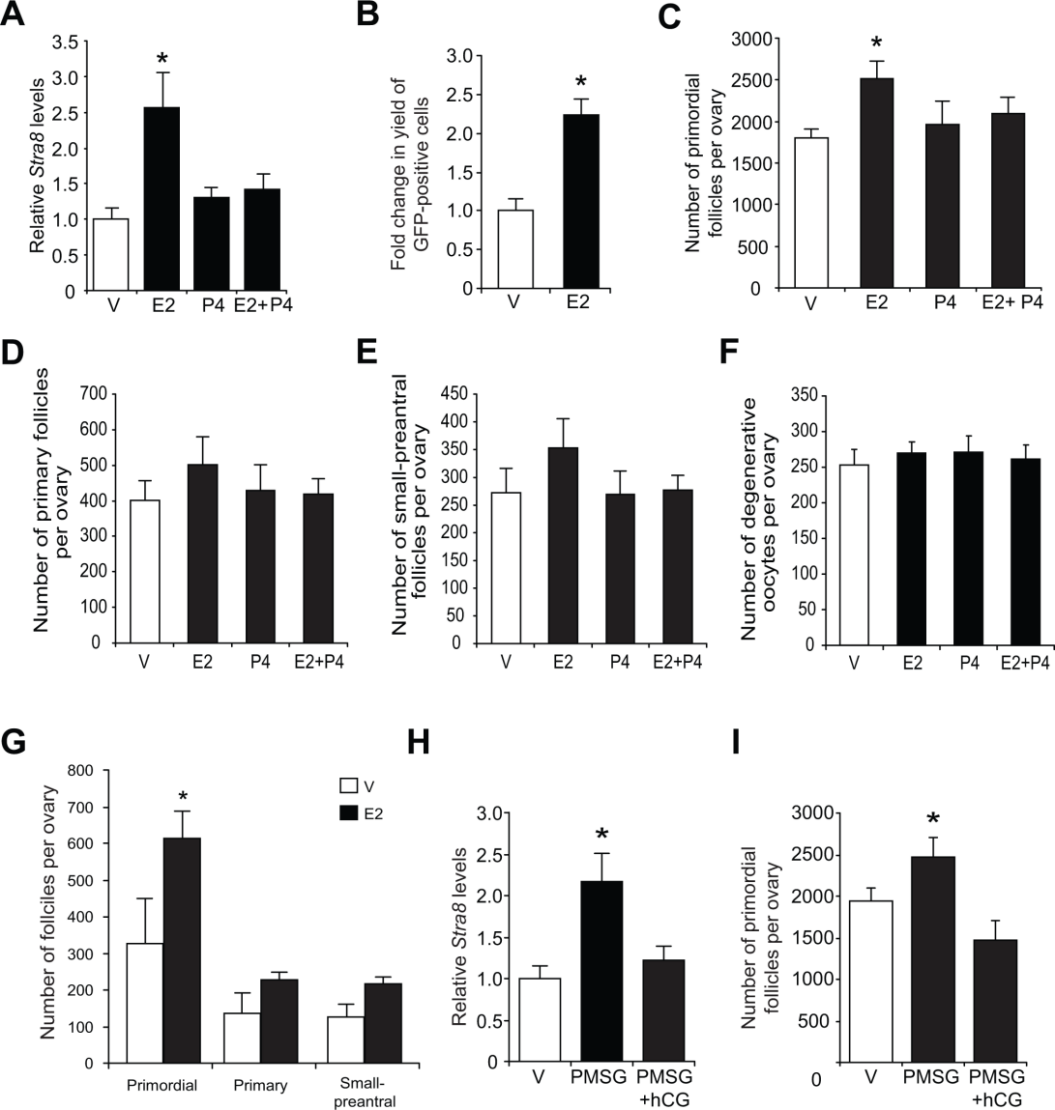
**Estrogen enhances ovarian *Stra8* expression and oogenesis *in vivo*.** (**A**) Changes in *Stra8* mRNA levels in ovaries of young adult WT mice 24 hours after injection of vehicle (V), E2 (0.5 mg/kg), P4 (100 mg/kg) or E2 plus P4 (mean ± SEM, n = 3 mice per group; *P<0.05). (**B**) Yield of GFP-positive germ cells from ovaries of young adult *pStra8-GFP* mice 24 hours after injection of V or E2 (0.5 mg/kg) (mean ± SEM, n = 3 mice per group; *P<0.05). (**C**–**F**) Numbers of primordial (**C**), recently growth-activated (primary; **D**) and early growing (small-preantral; **E**) follicles, and of degenerative oocytes (**F**), in ovaries of young adult WT mice 24 hours after injection of V, E2 (0.5 mg/kg), P4 (100 mg/kg) or E2 plus P4 (mean ± SEM, n = 8–10 mice per group; *P<0.05). (**G**) Primordial follicle numbers in ovaries of reproductively aged (8-month-old) WT mice 24 hours after injection of V or E2 (0.5 mg/kg) (mean ± SEM, n = 3 mice per group; *P<0.05). (**H**, **I**) Changes in *Stra8* mRNA levels (**H**) and primordial follicle numbers (**I**) in ovaries of young adult WT mice 46 hours after injection of PMSG (10 IU) followed by hCG injection (10 IU) 16 hours later (mean ± SEM, n = 5–7 mice per group; *P<0.05).

Consistent with the *in-vitro* modeling data using cultured OSCs (Figure 2C), injection of E2 into adult wild type mice elevated ovarian *Stra8* mRNA levels, and this response was abolished by co-injection of P4 (Figure 3A). In adult transgenic female mice expressing green fluorescent protein (GFP) under control of the mouse *Stra8* promoter (*pStra8-GFP*) [69], E2 treatment increased the number of GFP-positive cells obtained following FACS of dissociated ovaries (Figure 3B). Since GFP-expressing cells purified from ovaries of adult *pStra8-GFP* female mice represent a premeiotic germ cell population intermediate between OSCs and oocytes [29, 69], these findings draw a parallel between E2-induced transcriptional activation of the *Stra8* promoter in germ cells of adult mouse ovaries *in vivo* (Figure 3B) with outcomes observed using adult ovary-derived OSCs cultured *in vitro* (Figures 2A–2D).

In further keeping with *in-vitro* modeling data using cultured OSCs (Figure 2D), approximately 700 more oocytes were detected in the primordial follicle pool of adult female mice injected with E2 compared with age-matched, vehicle-treated controls (Figure 3C). Injection of P4 alone had no effect on oocyte numbers, but it abolished the E2-induced increase in oocyte numbers (Figure 3C), mirroring that observed using cultured OSCs as a model for oogenesis (Figure 2D). The opposing actions of E2 and P4 on oocyte dynamics in adult ovaries were restricted to the primordial follicle pool in that steroid treatment, alone or in combination, did not alter the number of growing or atretic follicles (Figures 3D–3F). In addition, E2 remained capable of increasing oocyte numbers in female mice at more advanced reproductive ages (Figure 3G).

To test if an elevation in endogenous E2 levels could reproduce the effects of exogenous E2 injection on oogenesis *in vivo*, we injected adult mice with pregnant mare serum gonadotropin (PMSG) to hyperstimulate ovarian follicle maturation and E2 secretion. Ovarian *Stra8* expression and primordial follicle numbers were significantly increased 48 hours after PMSG injection (Figures 3H and 3I). A subsequent injection of human chorionic gonadotropin (hCG), which triggers ovulation of PMSG-ripened follicles leading to the formation of P4-producing corpora lutea, resulted in a reduction in both *Stra8* expression and oocyte numbers to or below those levels observed prior to PMSG injection (Figure 3H and 3I). These outcomes reinforce the data obtained from studies of purified OSCs cultured with steroids *in vitro* (Figures 2C and 2D), and of mice injected with exogenous steroids *in vivo* (Figures 3A and 3C), collectively supporting the conclusion that P4 opposes the stimulatory actions of E2 on adult oogenesis.

### Estrogen-induced *Stra8* expression is functionally tied to *in-vivo* oogenesis

**Figure 4:**
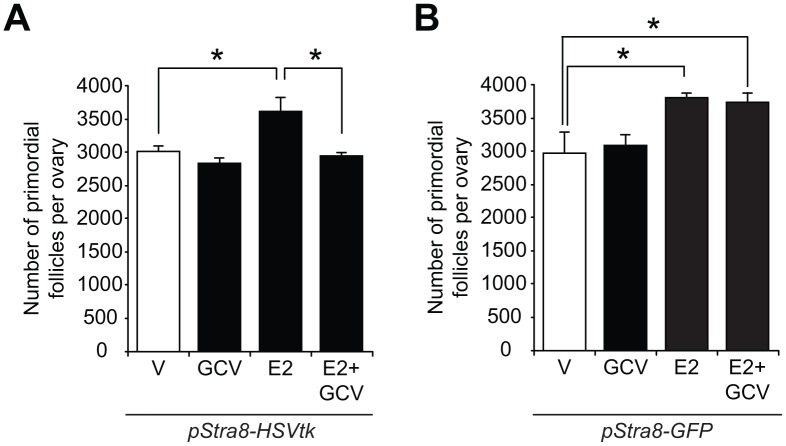
***Stra8* is involved in E2-induced oogenesis *in vivo*.** (**A**) Primordial follicle numbers in ovaries of young adult *pStra8-HSVtk* mice, pretreated with vehicle (V) or GCV (10 mg/kg) for 7 days, 24 hours after injection of E2 (0.5 m/kg) (mean ± SEM, n = 5–7 mice per group; *P<0.05). (**B**) Effects of vehicle (V) or E2 (0.5 mg/kg) injection on numbers of primordial follicles in ovaries of young adult *pStra8-GFP* mice pretreated without or with GCV (10 mg/kg) for 7 days (mean ± SEM, n = 5–7 mice per group; *P<0.05).

To determine the *in-vivo* relationship, if any, of E2-induced *Stra8* expression to changes in oogenesis, we examined the impact of temporally ablating *Stra8*-expressing germ cells in the absence or presence of exogenous E2 on oocyte numbers using a *Stra8* promoter-driven suicide gene transgenic mouse line (*pStra8-HSVtk*) [29]. This model enables targeted disruption of germ cells undergoing meiotic commitment associated with transcriptional activation of the *Stra8* gene, without directly affecting either OSCs (‘pre-Stra8’) or existing oocytes (‘post-Stra8’). Short-term treatment with the herpes simplex virus thymidine kinase (HSVtk) pro-drug, ganciclovir (GCV), did not affect primordial follicle numbers in adult *pStra8-HSVtk* mice over a 7-day period, consistent with past studies [29]; however, the oogenic response to exogenous E2 stimulation was completely abolished in *pStra8-HSVtk* mice pretreated with GCV for 7 days prior to E2 injection (Figure 4A). Specificity of this outcome was verified using adult *pStra8-GFP* mice as negative controls, in which GCV pretreatment for 7 days had no effect on E2-driven increases in primordial follicle numbers (Figure 4B). These data further support that E2 increases oocyte numbers through a pathway involving *Stra8* activation.

### Estrogen requires ERα to enhance *Stra8* expression and oogenesis

**Figure 5:**
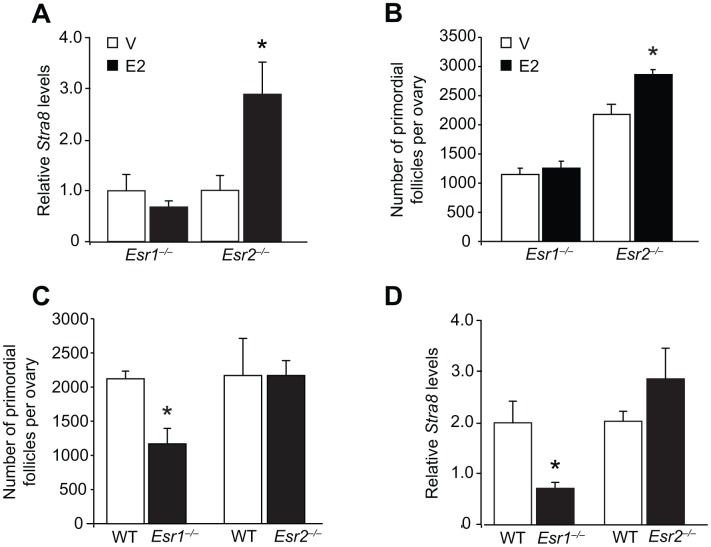
**Targeted disruption of the *Esr1* gene impairs E2-driven oogenesis during adulthood.** (**A**) Ovarian *Stra8* mRNA levels in young adult *Esr1^–/–^* and *Esr2^–/–^* mice (versus respective WT littermates) 24 hours after injection of vehicle (V) or E2 (0.5 mg/kg) (mean ± SEM, *n* = 5–6 mice per group; *P<0.05). (**B**) Primordial follicle numbers in ovaries of young adult *Esr1^–/–^* and *Esr2^–/–^* mice (versus respective WT littermates) 24 hours after injection of V or E2 (0.5 mg/kg) (mean ± SEM, *n* = 4–6 mice per group; *P<0.05). (**C**, **D**) Numbers of primordial follicles (**C**) and *Stra8* mRNA levels (**D**) in ovaries of *Esr1^–/–^* and *Esr2^–/–^* mice (versus respective WT littermates) at 2 months of age (mean ± SEM, *n* = 4–6 mice per group; *P<0.05).

Since OSCs express ERα (Figures 1A–1G), this may indicate an important role exists for this ER isoform in regulating OSC differentiation in response to E2. In agreement with this, we found that adult gene-mutant mice lacking ERα (*Esr1*-null, *Esr1^–/–^*), but not those lacking ERβ (*Esr2^–/–^*), failed to respond to exogenous E2 treatment with an elevation in either ovarian *Stra8* expression (Figure 5A) or oocyte numbers (Figure 5B). In addition, primordial follicle numbers in vehicle-treated *Esr1^–/–^* mice were lower than those of vehicle-treated *Esr2^–/–^* mice at the same ages (Figure 5B), indicating that ERα deficiency may be a critical regulator of oogenesis under physiological conditions. In agreement with this, by 2 months of age *Esr1^–/–^* mice had developed a premature ovarian insufficiency (POI) phenotype of significantly fewer oocytes when compared with their wild type (WT) female littermates (Figure 5C), along with reduced levels of ovarian *Stra8* expression (Figure 5D). No differences were detected in the numbers of primordial follicles or ovarian *Stra8* expression in age-matched adult WT versus *Esr2^–/–^* mice (Figures 5C and 5D).

### Female mice lacking ERα exhibit impaired postnatal oogenesis

**Figure 6:**
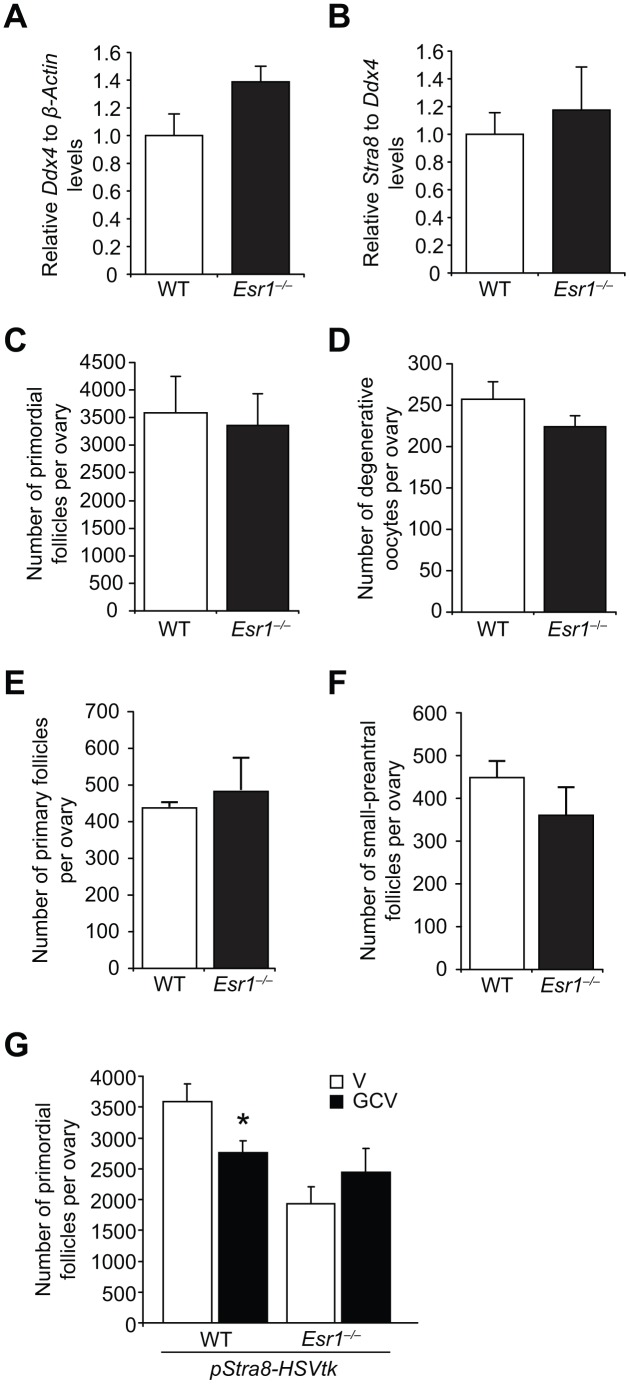
***Esr1*-null female mice exhibit impaired postnatal oocyte renewal.** (**A**, **B**) Expression of *Ddx4* (**A**; normalized to *β-actin*, indicative of the relative numbers of germ cells in whole gonads) and *Stra8* (**B**; normalized to *Ddx4*, indicative of the relative level of *Stra8* activation across the total germ cell pool) in e13.5 ovaries collected from WT and *Esr1^–/–^* female fetuses (mean ± SEM, n = 10 timed-pregnant female mice, with fetal ovaries of each genotype collected from each timed-pregnant dam serving as an independent replicate). (**C**) Primordial follicle numbers in ovaries of neonatal (5-day-old) *Esr1^–/–^* mice compared to WT littermates (mean ± SEM, n = 6 mice per group). (**D**) Numbers of degenerative oocytes in ovaries of young adult *Esr1^–/–^* mice compared to WT littermates (mean ± SEM, n = 4–6 mice per group). (**E**, **F**) Numbers of recently growth-activated (primary; **E**) and early growing (small-preantral; **F**) immature follicles in ovaries of *Esr1^–/–^* mice, compared to WT littermates, at 2 months of age (mean ± SEM, n = 4–6 mice per group). (**G**) Primordial follicle numbers in ovaries of young adult *pStra8-HSVtk;WT* and *pStra8-HSVtk*;*Esr1^–/–^* mice treated with vehicle (V) or GCV (10 mg/kg) for 21 days (mean ± SEM, n = 5–6 mice per group; *P<0.05).

There are four possible explanations for the POI phenotype in young adult *Esr1^–/–^* mice: *i*) abnormalities in primordial germ cell (PGC) function during fetal development that result in fewer oocytes endowed at birth; *ii*) endowment of a normal quota of oocytes at birth that is then depleted more quickly through atresia; *iii*) endowment of a normal quota of oocytes at birth that is then depleted more quickly through primordial follicle growth activation; or, *iv*) attenuation of postnatal oocyte renewal caused by disruption of E2-initiated signaling coupled to OSC differentiation. A developmental defect in PGC function was tested by two approaches. First, we found that expression levels of the germ cell marker, *Ddx4*, and the meiotic marker, *Stra8*, were comparable in ovaries of WT and *Esr1^–/–^* fetuses at embryonic (e) day 13.5 (e13.5), when peak oocyte numbers are formed in developing ovaries (Figures 6A and 6B). These results, which indirectly suggest that PGC number (*Ddx4*) and function (*Stra8* expression, meiotic entry) are unaltered by ERα deficiency during embryonic ovarian development, were supported by direct evidence that the number of oocytes endowed in the ovaries of neonatal mice were comparable in WT and *Esr1^–/–^* females (Figure 6C). In addition to being provided with a normal quota of oocytes at birth, *Esr1^–/–^* mice also showed no difference in the levels of postnatal oocyte loss through atresia (Figure 6D), or any change in the numbers of immature growing follicles as a measure of primordial follicle growth activation (Figures 6E and 6F), when compared with their WT female littermates.

These outcomes left us to consider the fourth possibility, namely that attenuated postnatal oocyte renewal drives emergence of the POI phenotype in *Esr1^–/–^* females as the mice transition from neonatal life (normal oocyte endowment) to adulthood (lower oocyte reserve). Our observation that ovarian *Stra8* expression was reduced in adult *Esr1^–/–^* females compared with age-matched WT littermates (Figure 5D) provided the first clue that impaired differentiation of OSCs may be occurring in the absence of functional ERα. To directly evaluate this, we introduced the *pStra8-HSVtk* allele into *Esr1^–/–^* mice, which would allow us to assess if *Esr1^–/–^* mice exhibit defects in oogenesis by quantifying the impact of targeted ablation of *Stra8*-expressing germ cells on oocyte dynamics in the absence or presence of ERα. Adult *pStra8-HSVtk* mice with functional ERα (*pStra8-HSVtk*;WT) exhibited a reduced primordial follicle pool after 21 days of GCV exposure (Figure 6G), due to a progressive impairment in new oocyte input that normally offsets natural oocyte loss over the 3-week treatment period [29]. In contrast, treatment of *pStra8-HSVtk*;*Esr1^–/–^* mice with GCV for 21 days had no effect on oocyte numbers versus vehicle-treated *pStra8-HSVtk*;*Esr1^–/–^* mice (Figure 6G). This insensitivity of *pStra8-HSVtk*;*Esr1^–/–^* mice to GCV exposure is consistent with minimal, if any, ongoing Stra8-driven oocyte renewal in ERα-deficient females.

### Actions of ERα on *Stra8* expression are specific to adult female gonads

**Figure 7:**
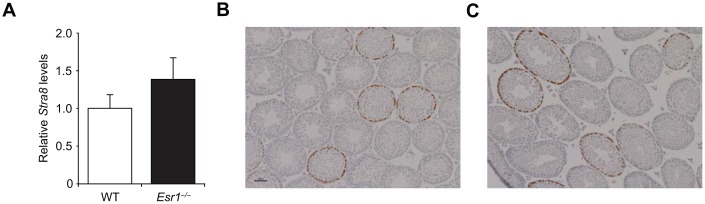
**Lack of effect of *Esr1* gene disruption in testes of adult male mice.** (**A**) Quantitative analysis of *Stra8* mRNA levels in testes of wild type (WT) and *Esr1*^–/–^mice at 3 months of age (mean ± SEM; n = 3 mice per group). (**B**, **C**) Histological appearance of the testes of WT (**B**) and *Esr1*^–/–^ (**C**) mice at 3 months of age, after fixation and analysis by immunohistochemistry for Stra8 expression (brown immunoreaction product against a blue hematoxylin counterstain).

**Figure 8:**
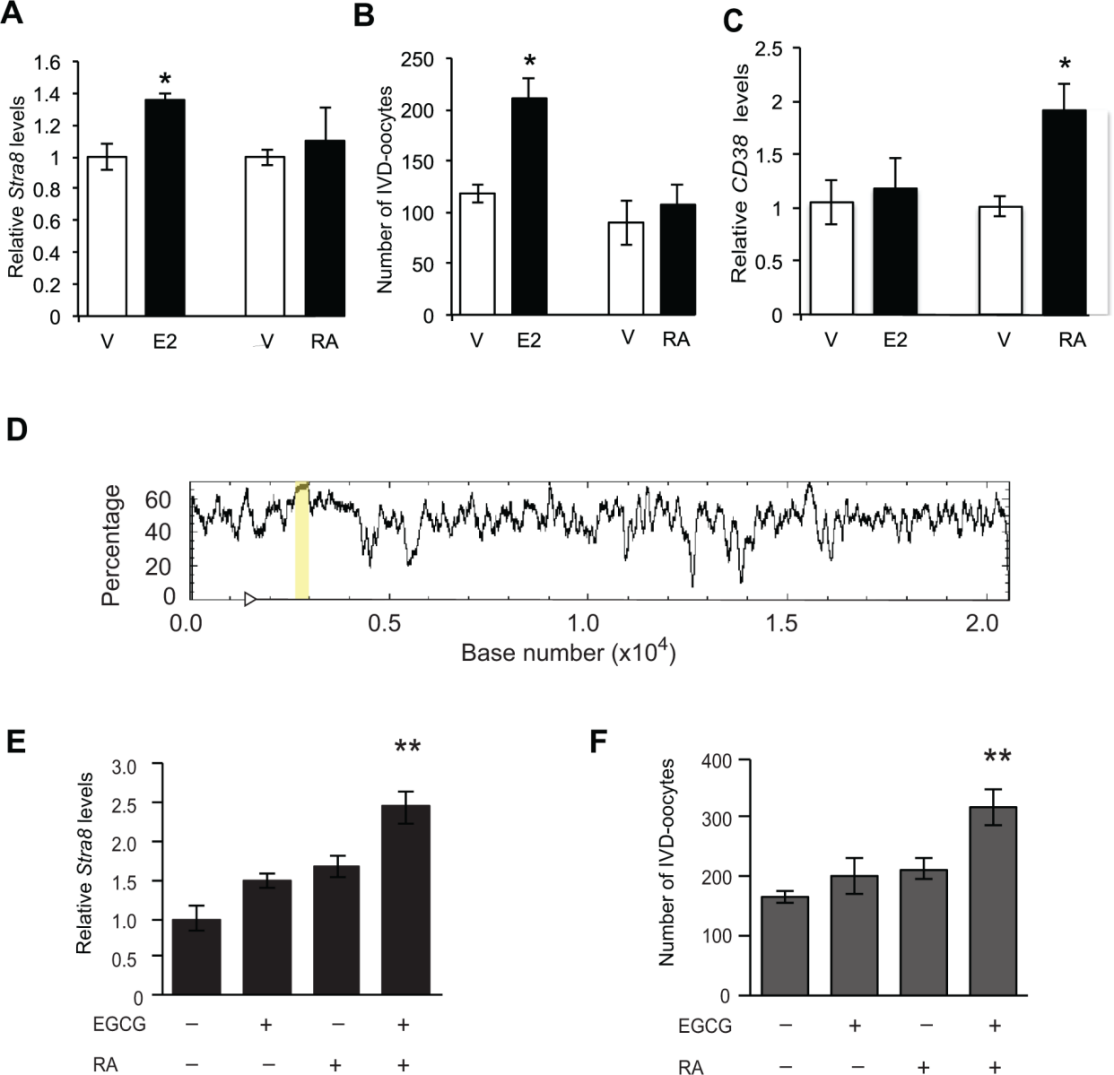
**Enhancement of OSC differentiation by RA requires repression of DNA methyltransferase activity.** (**A**, **B**) Levels of *Stra8* mRNA (**A**) and numbers of IVD-oocytes generated (**B**) in cultures of OSCs treated with vehicle (V), E2 (10 nM) or RA (2 μM) for 8 hours (mean ± SEM, n = 3 independent cultures; *P<0.05). (**C**) Levels of *CD38* mRNA in OSCs cultured with vehicle (V), E2 (10 nM) or RA (2 μM) for 8 hours (mean ± SEM, n = 3 independent cultures; *P<0.05). (**D**) *In-silico* analysis of the *Stra8* genomic sequence (transcription start site indicated by arrowhead), identifying a CpG island spanning 386-bp (highlighted by the yellow bar). (**E**, **F**) Levels of *Stra8* mRNA (**E**) and numbers of IVD-oocytes generated (**F**) in cultures of OSCs treated without or with 2-μM RA for 8 hours after a 4-hour pretreatment without or with 50-μM EGCG (–, without; +, with) (mean ± SEM, n = 6 independent cultures; **P<0.01).

Given the well-established role of RA-mediated signaling in driving *Stra8* expression and meiotic commitment in germ cells of both embryonic ovaries and adult testes [58–63], we next explored if disruption in *Stra8* expression and gametogenesis resulting from an absence of ERα in adult females (Figure 5) also occurs in adult *Esr1^–/–^* male mice. Assessment of testicular *Stra8* expression and morphology revealed no discernible differences between wild type and *Esr1^–/–^* males (Figure 7), indicating that the role of E2 signaling through ERα in controlling germline stem cell activity is apparently restricted to females. Since, however, the absence of functional ERα had no discernible consequences on the ability of embryonic ovaries to generate a normal quota of oocytes at birth (Figure 6C), this left us with the question of whether RA, which is the principal *in-vivo* driver of embryonic oogenesis [58–63], can induce differentiation of adult ovary-derived OSCs. In contrast to the stimulatory effects of E2 on *Stra8* expression and IVD-oocyte formation in cultured OSCs, treatment of OSCs in parallel with RA failed to alter either endpoint (Figures 8A and 8B). As an assurance control for RA sensitivity and dosing, OSCs exposed to RA did exhibit a significant increase in expression of *CD38* (Figure 8C), which is a widely known transcriptional target for RA-driven gene expression [70]. These latter results indicate that a developmental specificity apparently exists in the *in-vivo* cues responsible for activating germ cell meiotic commitment in embryonic versus adult female gonads.

To gain initial insights into a potential mechanism underlying this change in the control of *Stra8* expression in female germ cells from embryonic to postnatal life, we directed our attention to epigenetics. This seemed logical given that OSCs failed to increase *Stra8* expression when exposed to RA, but exhibited RA responsiveness if expression of a different RA-target gene (*CD38*) was evaluated in the same cell population. Additionally, earlier studies have implicated epigenetic status as a key regulator of *Stra8* expression and germ cell meiotic commitment in mammalian ovaries [67]. Through *in-silico* analysis, we identified a 386-bp CpG island within the first intron of the *Stra8* gene (Figure 8D), suggesting that DNA methylation may play a role in regulating sensitivity of *Stra8* to different transcriptional activators. In keeping with this, pretreatment of OSCs with the DNA (cytosine-5)-methyltransferase 1 (Dnmt1) inhibitor, epigallocatechin-3-gallate (EGCG) [71], for 4 hours prior to the addition of RA resulted in enhanced *Stra8* expression and IVD-oocyte formation in RA-treated OSC cultures (Figure 8E and 8F).

### Pharmacologic disruption of E2 signaling causes reversible oogenic failure

**Figure 9:**
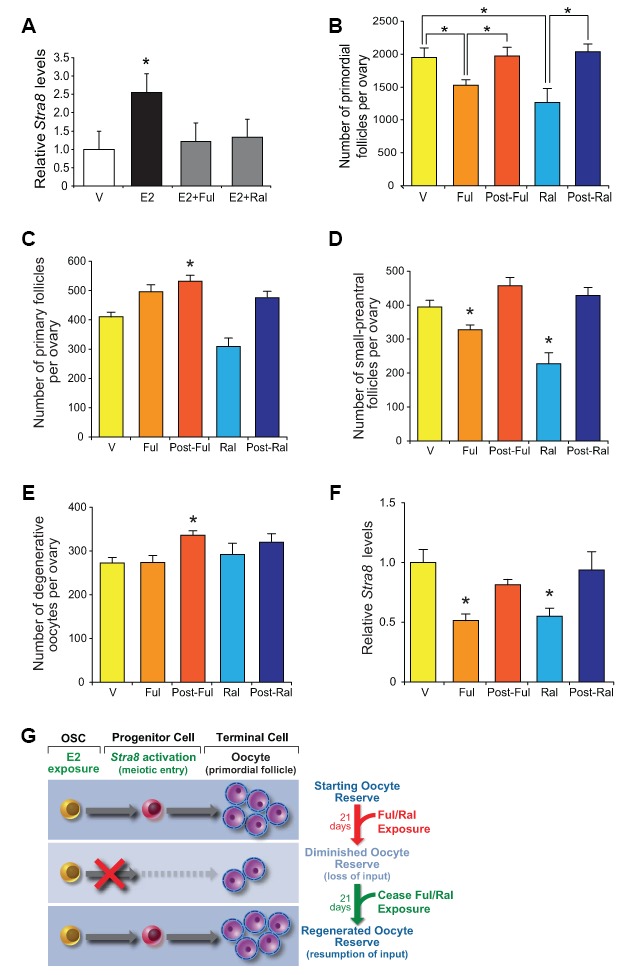
**Pharmacological suppression of ER signaling reversibly impairs oogenesis during adulthood.** (**A**) Changes in *Stra8* mRNA levels in OSCs cultured with vehicle (V), E2 (10 nM), E2 plus fulvestrant (Ful, 10 nM; E2+Ful), or E2 plus raloxifene (Ral, 10 nM; E2+Ral) for 24 hours (mean ± SEM, n = 3 independent cultures; *P<0.05). (**B**) Primordial follicle numbers in ovaries of young adult WT mice treated with V, Ful (10 mg/kg), or Ral (20 mg/kg) for 21 days, and 21 days after ceasing Ful or Ral treatment (post-Ful or post-Ral, respectively) (mean ± SEM, n = 7–13 mice per group; *P<0.05). (**C**–**E**) Numbers of recently growth-activated (primary; **C**) and early growing (small-preantral; **D**) immature follicles, and of degenerative oocytes (**E**), in ovaries of WT mice treated as described in panel **B** (mean ± SEM, n = 7–13 mice per group; *P<0.05). (**F**) Changes in ovarian *Stra8* mRNA levels in WT mice treated as described in panel **B** (mean ± SEM, n = 7–13 mice per group; *P<0.05). (**G**) Schematic depiction of how reversible ER antagonists likely alter oocyte dynamics in adult ovaries by transiently disrupting endogenous E2-promoted OSC differentiation into new oocytes, yielding a net decline in total oocyte numbers due to attenuated input that then fully recovers after ceasing ER antagonist exposure.

To reinforce our genetic studies on the role of E2-initiated signaling in postnatal oogenesis, in a final set of experiments we tested the effects of two widely used, reversible ER modulators on *Stra8* expression and oocyte numbers. While fulvestrant is considered a pure ER antagonist that works by promoting ER degradation [68], raloxifene can have either ER agonistic or antagonistic activity depending on the target tissue or cell type [72]. In cultured OSCs, fulvestrant and raloxifene efficiently inhibited E2-induced *Stra8* expression (Figure 9A), indicating that both drugs work directly on OSCs to antagonize ER-mediated signaling. *In vivo*, adult mice treated for 21 days with either fulvestrant or raloxifene exhibited a significantly diminished primordial oocyte (follicle) pool (Figure 9B), which could not be attributed to accelerated growth activation to more advanced follicle stages or to increased loss through atresia (Figures 9C–9E). After ceasing drug treatment, the primordial follicle pool spontaneously regenerated over a subsequent 21-day period back to the size observed prior to the initiation of drug exposure (Figure 9B). Changes in oocyte numbers in response to ER antagonist exposure (reduced) and removal (regenerated) were paralleled by similar changes in ovarian *Stra8* expression (Figure 9F). These data, indicative of a resumption of E2-driven oogenesis to replenish the depleted follicle pool once antagonism of ER signaling was removed (Figure 9G), mirror the reversible oogenic failure reported to occur in a genetic mouse model of targeted and reversible premeiotic germ cell ablation [29].

## DISCUSSION

Recent evidence from two different genetic studies with mice demonstrating that postnatal oocyte formation is a physiologically important aspect of adult ovarian function and fertility in mammals [29, 55] provides a strong impetus to identify the endocrine regulators and molecular signaling pathways responsible for coordination of OSC differentiation *in vivo*. Prior investigations with mice have shown that bone morphogenetic protein 4 (BMP4), a known regulator of embryonic PGC specification [73, 74], can enhance meiotic gene expression and oogenesis in cultured OSCs through SMAD1/5/8 signaling [75]. Additionally, more recent studies of cultured mouse and human OSCs indicate that extracellular matrix proteins influence the differentiation of these cells *in vitro* in a species-specific manner [76]. However, little is known of the *in-vivo* cues used by OSCs to support oocyte formation during adult life, and if changes in these cues with age may contribute to a loss of oogenic support of the ovaries. By employing several complementary genetic and pharmacologic tools with *in-vitro* and *in-vivo* models of OSC function and oocyte formation, our experimental outcomes have uncovered a novel and functionally indispensable role for E2-ERα–initiated signaling in controlling OSC differentiation and *de-novo* oogenesis in adult mouse ovaries.

Prior studies of mouse fetal gonads have reported that PGCs express ERα, and that E2-dependent activation of this receptor can promote embryonic germ cell proliferation [77]. Additionally, this study also concluded that the ability of E2 to enhance PGC proliferation may be mediated through non-genomic actions of E2 in PGCs involving activation of the KIT receptor signaling cascade [77]. Studies of human fetal gonads have similarly reported expression of ER in PGCs around mid-gestation [78]. Additionally, both isoforms of ER can be detected in human oocytes as follicles are being formed during the second half of gestation, suggesting that ER signaling may play a role in human folliculogenesis prior to birth [79]. However, none of these studies reported effects of E2 or ER signaling on the activation of embryonic germ cell meiosis or oogenesis. Accordingly, these data from studies of PGCs in embryonic gonads starkly contrast our observations from analysis of OSCs in adult ovaries, the latter of which show that E2 does not affect OSC proliferation but instead serves as a novel driver of meiotic commitment and oogenesis.

These findings have important implications on several fronts. For example, it was previously reported that activity of hematopoietic stem cells in mice is also regulated by cyclic production of E2 from the ovaries during adulthood [80]. When taken with our observations, these findings collectively highlight the existence of a broad *in-vivo* function for ovarian-derived steroids in controlling the activity and differentiation of adult stem cell populations both inside and outside of the gonads. Interestingly, however, the role of E2-mediated signaling through ERα in the regulation of germline stem cell activity appears restricted to females, in that we found *Stra8* expression and spermatogenesis in adult testes were unaffected by disruption of the *Esr1* gene. As such, these data have also identified one of the first signaling pathways required for driving the meiotic differentiation of female, but not male, germ cells. The apparent sex-specific role of E2-ERα–initiated signaling in supporting gametogenesis may reflect the critical importance of E2 to female reproductive function and health, and perhaps the consequences of a loss of ovarian E2 production with age as women approach the menopause. Regarding the latter, recent studies have reported that OSCs persist in the ovaries of mice and women, even after the time of natural age-related ovarian failure [29, 37, 41]. Interestingly, OSCs in aged ovaries retain the ability to undergo differentiation into oocytes if provided with appropriate cues, through either *in-vitro* culture [37, 41] or *in-vivo* heterochronic transfer into a young ovarian microenvironment [81]. Thus, a progressive impairment in the ability of OSCs to support oogenesis with age, as demonstrated by suicide gene-based technologies [29], may reflect changes in the availability of extrinsic factors, such as E2, needed for *in-vivo* maintenance of OSC differentiation. Future studies of human OSCs, which show a steroid receptor expression pattern similar to that seen in mouse OSCs (D.C. Woods and J.L. Tilly, unpublished observations), should offer additional insights into this possibility in women.

As mentioned earlier, it is also of interest that the ability of E2-ERα–initiated signaling to drive *Stra8* expression and oogenesis appears specific for OSCs in adult ovaries since *Esr1* gene disruption had no impact on fetal oogenesis, as reflected by the establishment of a neonatal pool of oocyte-containing follicles in *Esr1*-null females which was no different than that of WT siblings. Prior studies have shown that prenatal oogenesis, which generates the oocytes found in primordial follicles of neonatal ovaries, is driven by RA-initiated induction of *Stra8* expression in PGCs of embryonic gonads [58–63]. Diverging from this widely held paradigm of germ cell meiotic commitment, our data indicate that the differentiation of mitotically active germ cells in adult mouse ovaries apparently no longer depends on RA, but instead has changed to the use of E2-ERα as a primary signaling mechanism for supporting oogenesis. Although the underlying basis for this developmental switch in cues in the female germ line remains unknown at present, past studies have reported that epigenetic events may be key determinants of germ cell meiotic commitment in mammalian ovaries [67, 82]. Other studies have reported that specific epigenetic events may also be involved in conveying OSC identity, unipotency and function [83]. In alignment with these findings, we observed that OSCs cultured in the presence of a Dnmt1 inhibitor gained responsiveness to RA exposure with respect to inducible *Stra8* expression. Future experiments to further delineate the molecular mechanisms responsible for the developmental divergence in meiotic-initiating cues between PGCs and OSCs will be of interest to pursue. In conclusion, these data collectively add to the growing body of work to define the properties, regulation and function of OSCs in adult mammalian ovaries [42, 43]. Additionally, this work opens the possibility that the well described changes in ovarian E2 production as females age may underlie, at least in part, OSC dysfunction and a corresponding loss of oogenic support as mechanisms that contribute to aging-associated ovarian failure. Studies of how E2 interacts with other pathways already tied to OSC function, such as BMP4 signaling [75] and extracellular matrix proteins [76], and whether E2 affects pathways in addition to Stra8 that have been linked to meiosis [84], should offer a more complete picture of how changes in ovarian-intrinsic and -extrinsic factors play into reproductive aging.

## MATERIALS AND METHODS

### Animals and treatments

Wild-type C57BL/6 mice were obtained from Charles River Laboratories. Heterozygous mutant mice with a targeted disruption of *Esr1* (*B6.129P2-Esr1tm1Ksk/J*; stock number: 004744) or *Esr2* (*B6.129P2-Esr2tm1Unc/J*; stock number: 004745) were obtained from the Jackson Laboratory and used to set up breeding colonies for direct comparative studies of WT and homozygous-null littermates. All mice were backcrossed to congenic C57BL/6 prior to use. Transgenic knock-in mice with *GFP* or *HSVtk* expression driven by a 1.5-kb fragment of the mouse *Stra8* promoter (*pStra8-GFP* or *pStra8-HSVtk*, respectively) were generated and maintained as described [29, 69]. All steroid injections were delivered via the intraperitoneal cavity, and dosing strategies were based on extensive prior mouse studies using steroid injections as a treatment protocol. Stock solutions of E2 (MilliporeSigma) and P4 (MilliporeSigma) were prepared in ethanol prior to dilution in sesame oil as a vehicle for injection of each steroid (E2, 0.5 mg/kg body weight; P4, 100 mg/kg body weight). Ganciclovir (Roche) was dissolved in sterile water at 10 mg/ml and then diluted in sterile 1X-concentrated phosphate-buffered saline (PBS) for once daily intraperitoneal injections (10 mg/kg body weight for the indicated number of days) [29]. Gonadotropins (MilliporeSigma) were prepared in 1X-PBS for subcutaneous injections (PMSG, 10 IU per mouse; hCG, 10 IU per mouse). Fulvestrant (MilliporeSigma) was dissolved in ethanol at 10 mg/ml as stock prior to further dilution to 1 mg/ml in sesame oil for subcutaneous injection every 2 days at 10 mg/kg body weight. Raloxifene (MilliporeSigma) was dissolved in 50% DMSO (vol:vol), 40% 1X-PBS (vol:vol) and 10% ethanol (vol:vol) at 13.3 mg/ml, and then injected subcutaneously at 20 mg/kg body weight every day for the duration of the treatment protocol. All animal procedures were reviewed and approved by the institutional animal care and use committees of Massachusetts General Hospital and Northeastern University.

### Oocyte counts by histomorphometry

Complete and serially sectioned mouse ovaries were processed for histomorphometry-based quantification of the numbers of healthy or degenerative (atretic) oocyte-containing follicles at the indicated stages of development, as detailed [29]. This protocol has been rigorously validated for reproducibility and accuracy in identification and direct quantification of oocytes at the indicated stages of development [29].

### Isolation and culture of OSCs

Oogonial stem cells were isolated by fluorescence-activated cell sorting (FACS) from dispersed ovaries of mice at 2 months of age based on externalized expression of the C-terminus of Ddx4 in viable cells and established as actively dividing germ cell cultures without somatic feeder cells [24, 50]. Purified mouse OSCs propagated under these conditions spontaneously generate IVD-oocytes for up to 72 hours after passage until confluence is regained, and the number of IVD-oocytes generated by a fixed number of OSCs seeded per well remains relatively constant over successive passages [24, 29, 37, 50, 75, 76]. Accordingly, IVD-oocyte formation can be used as a very reliable and rapid bioassay for identification of factors that affect OSC differentiation [29, 75, 76]. In some experiments, OSCs (passages 28–34) were seeded into 24-well tissue culture plates (2.5 X 10^4^ cells/well) in OSC culture medium containing charcoal-stripped 10% fetal bovine serum (FBS) (Thermo Fisher), acclimated for 24 hours, and then exposed to vehicle (ethanol, 0.1% final), E2 (10 nM), P4 (2 μM), E2 plus P4, or E2 (10 nM) in the absence or presence of raloxifene (10 nM) or fulvestrant (10 nM) for up to 72 hours. Concentrations of all treatments used were based on extensive prior studies of these hormones and compounds *in vitro* as well as on in-house empirical testing (not shown). In other experiments designed to directly compare the effects of E2 and RA, OSCs (passages 30–35) were cultured in hormone-free OSC medium composed of phenol red-free minimum essential medium-α (Thermo Fisher), charcoal-stripped 10% FBS (Thermo Fisher), 20 μg/ml transferrin (MilliporeSigma), 5 μg/ml insulin (MilliporeSigma) and 60 μM putrescine (MilliporeSigma) for 7 days, and then seeded into 6-well (1.25 X 10^5^ cells/well; gene expression) or 24-well (2.5 X 10^4^ cells/well; IVD-oocyte formation) tissue culture plates. After 24 hours of acclimation, the cells were exposed to each vehicle (0.1% ethanol or 0.2% DMSO), E2 (10 nM) or RA (2 μM) for up to 24 hours. Changes in gene expression (*Stra8*, *CD38*) were then assessed, and numbers of IVD-oocytes generated were determined by direct visual counts [24, 29, 37, 50, 75, 76].

### Gene expression analysis

Total RNA was extracted using Tri-Reagent (MilliporeSigma) or RNAzol^®^ RT (MilliporeSigma), treated with DNase1 (Thermo Fisher) to remove genomic DNA, and reverse transcribed (1-μg of total RNA per sample) using either Superscript III (Thermo Fisher) or RevertAid (Thermo Fisher) reverse transcriptase along with oligo-dT primers into cDNA. In some experiments designed to assess if a specific mRNA transcript was simply present or not, amplification of target gene sequences was performed by conventional PCR using primers specific for each gene (Table 1). All products were sequenced for identity confirmation. For quantitative comparative analysis of the relative levels of specific mRNA transcripts across samples, qPCR was performed using a Cepheid Smart Cycler II Automated Real-time PCR System, along with primers specific for each target sequence (Table 1). Data were analyzed by the ΔΔC_t_ method of relative quantitation.

**Table 1:** Primers used for conventional and real-time (quantitative, q) PCR analysis of gene expression.

**Conventional PCR analysis**
β-actin, NCBI Gene ID 11461: forward, 5'-GATGACGATATCGCTGCGCTG-3'; reverse, 5'-GTACGACCAGAGGCATACAGG-3'
ERα, NCBI Gene ID 13982: forward, 5’-CAGGTGCCCTACTACCTGGA-3’; reverse, 5’-CCTGAAGCACCCATTTCATT-3’
ERβ, NCBI Gene ID 13983: forward, 5’-CAATCATCGCTTCTCTATGCAG-3’; reverse, 5’-TTTTACGCCGGTTCTTGTCTAT-3’
PR, NCBI Gene ID 18667: forward, 5’-GGTGGAGGTCGTACAAGCAT-3’; reverse, 5’-AAATTCCACAGCCAGTGTCC-3’
Stra8, NCBI Gene ID 20899: forward, 5'-GCCAGAATGTATTCCGAGAA-3'; reverse, 5'-CTCACTCTTGTCCAGGAAAC-3'
**Real-time PCR analysis**
β-actin, NCBI Gene ID 11461: Invitrogen FAM-labeled primer set 101M-01 for mouse and rat β-actin, used as a sample loading control
β-2-microglobulin, NCBI Gene ID 12010; forward, 5’-TTCTGGTGCTTGTCTCACTGA-3’; reverse, 5’-CAGTATGTTCGGCTTCCCATTC-3’, used as a sample loading control
CD38, NCBI Gene ID 12494: forward 5’-TTGCAAGGGTTCTTGGAAAC-3’; reverse, 5’-CGCTGCCTCATCTACACTCA-3’
Ddx4, NCBI Gene ID 13206: forward 5’-GCAGAGATGTTCAGCAGACG-3’; reverse, 5’-ATCGCTCTGCCAGTATTTCC-3’
Gapdh, NCBI Gene ID 14433: TaqMan primer set for mouse Gapdh (Assay ID Mm99999915_g1)
Stra8 (Figures 1, 2–5, 7), NCBI Gene ID 11461: FAM-labeled primer set MLUX3312362 for mouse Stra8 (Invitrogen)
Stra8 (Figure 6) NCBI Gene ID 11461: TaqMan primer set for mouse Stra8 (Assay ID Mm00486473_m1)

### DNA methylation analysis

To identify potential sites of epigenetic regulation of *Stra8*, the reference genomic sequence (NCBI Gene ID: 20899) was analyzed using the Genome Browser CpG Islands Tracks [85]. This analysis identified a region within the first exon of the *Stra8* gene (chr6:34921372-34921757) meeting the following criteria of a CpG island: GC content 50% or greater (66.3%), length >200-bp (386-bp), and ratio >0.6 of observed CG dinucleotides to the expected number (0.81). For cell studies, 24 hours after plating 2.5 X 10^4^ OSCs in plastic 24-well tissue culture plates containing 0.5 ml of OSC culture medium, the cells were pretreated with 50**-**μM EGCG (Tocris Bioscience) for 4 hours prior to treatment with RA (2**-**μM) for an additional 20 hours. The culture medium was then evaluated for the number of IVD-oocytes, while adherent cells were collected in RNAzol^®^ RT for RNA isolation and reverse transcription with RevertAid. Quantitative PCR analysis of *Stra8* mRNA levels was then performed using *Stra8*-specific oligonucleotide primers (forward: 3’-GAGGCCCAGCATATGTCTAAC-5’; reverse: 3’-GCTCTGGTTCCTGGTTTAATG-5’), along with primers specific for *beta-2-microglobulin* as a reference gene (forward: 3’-TTCTGGTGCTTGTCTCACTGA-5’; reverse: 3’-CAGTATGTTCGGCTTCCCATTC-5’), using SYBR Green (Thermo Fisher). Data were analyzed by the ΔΔC_t_ method of relative quantitation.

### Western blot analysis

Total proteins were isolated from cultured OSCs (passage 30) or ovaries (2.5-month-old mice; used a positive controls) in RIPA buffer [10 mM Tris-HCl (pH 7.4), 150 mM NaCl, 1% Triton X-100, 1.0% sodium deoxycholate, 0.1% SDS, 1.0 mM EDTA] supplemented with a protease inhibitor cocktail (Roche). Lysates were centrifuged at 14,000 x *g* for 10 min at 4°C, and protein concentrations in supernatants were determined using the BCA protein assay (Thermo Fisher). Twenty μg of protein from each sample were mixed with 2X-concentrated Laemmli Sample Buffer (BioRad) and then denatured for 10 min at 70°C. Proteins were resolved by sodium dodecyl sulfate (SDS)-polyacrylamide gel electrophoresis (SDS-PAGE) using 7.5% reducing-denaturing gels and transferred to nitrocellulose membranes. Blots were probed with antibodies against ERα (ab32063, 1:1,000 dilution; Abcam), ERβ (PA1311, 1:1,000 dilution; Thermo Fisher), PR (PA568778, 1:1,000 dilution; Thermo Fisher) or β-actin (MS-1295-P, 1:10,000 dilution; Lab Vision/NeoMarkers), washed and reacted with a 1:400 dilution of TidyBlot horseradish peroxidase-conjugated detection reagent (BioRad). Detection was performed with the Clarity™ Western ECL Substrate (BioRad) using a ChemiDoc imaging system (BioRad).

### Flow cytometric analysis

Cultured OSCs (passage 30) were fixed in 3.7% formaldehyde for 15 min at 22°C (room temperature). Following fixation, cells were permeabilized in 1X-PBS containing 0.1% Triton-X for 5 min at room temperature, and washed in 1X-PBS containing 0.01% Triton-X. Cells were then blocked in 10% normal goat serum (MilliporeSigma) for 30 min at room temperature, washed and incubated with the following conjugated antibodies: rabbit anti-ERα (ab32063, Abcam) conjugated to allophycocyanin (APC, Abcam), rabbit anti-DDX4 (ab13840, Abcam) conjugated to AlexaFluor 488 (Thermo Fisher), or a normal rabbit IgG isotype control conjugated with a matched fluorophore (Thermo Fisher). The cells were then analyzed by flow cytometry using a FACSAria-III (Becton, Dickinson and Company) and gated against unlabeled cells (those incubated with the normal rabbit IgG isotype control) as a baseline.

### ChIP-PCR analysis

Cell lysates were processed using the EZ-ChIP^TM^ kit (MilliporeSigma) with a mouse monoclonal anti-ERα antibody (17-603, MilliporeSigma) for immunoprecipitation, as per the manufacturer’s protocol. Precipitated soluble chromatin was then subjected to PCR to amplify a 149-bp region of the mouse *Stra8* promoter containing a consensus ERE sequence using the following primers: forward, 5’-CAAGTGACCTCCGTTTAACCTC-3’; reverse, 5’-GAGAAAGGAAAGCAAGCAAAAG-3’. To confirm specificity of ERα binding with the *Stra8* promoter, 2 X 10^6^ OSCs at passage 30 were seeded onto 10-cm^2^ tissue culture plates and acclimated for 24 hours. The cells were then exposed to vehicle or the pure ER antagonist, fulvestrant (10 nM), for 30 min prior to the addition of E2 (10 nM). After 4 hours (empirically determined to be the peak time for ERα interaction with the *Stra8* promoter in the presence of E2 alone; Figure 2A), the cells were collected and processed for ChIP-PCR analysis as described above.

### Immunohistochemistry

Freshly collected tissues were fixed in 4% paraformaldehyde, embedded in paraffin, and sectioned for analysis using a rabbit polyclonal antibody against Stra8 (ab49602, Abcam). Detection was performed using biotin-conjugated anti-rabbit IgG as secondary antibody for streptavidin-horseradish peroxidase–based 3,3’-diaminobenzidine detection (MilliporeSigma). Images were captured using a Nikon ECLIPSE TE2000-S microscope.

### Experimental replication and data analysis

All experiments were independently replicated at least three times, using different mice, tissues from different mice, or cells for each biological replicate. Where possible, assignment of mice to experimental groups was made randomly. Quantitative data from the experimental replicates for each study design were combined (mean ± SEM) and analyzed by one-way ANOVA followed by Student’s *t*-test for statistical differences (set at P<0.05). Qualitative images presented are representative of the outcomes obtained in the experimental replicates.
